# Parental Stress and Chinese American Preschoolers’ Adjustment: The Mediating Role of Parenting

**DOI:** 10.3390/bs13070562

**Published:** 2023-07-06

**Authors:** Suqing Wang, Charissa S. L. Cheah, Xiaoli Zong, Huiguang Ren

**Affiliations:** Department of Psychology, University of Maryland, Baltimore County, Baltimore, MD 21250, USA; swang7@umbc.edu (S.W.); zxiaoli1@umbc.edu (X.Z.); hgren@umbc.edu (H.R.)

**Keywords:** socio-emotional and behavioral adjustment, Chinese American preschoolers, general/contextual stress, parenting stress, psychologically controlling parenting practices, warmth practices

## Abstract

Family contexts, such as parental stress and parenting practices, play critical roles in preschoolers’ adjustment. However, these processes have been understudied in Chinese American families. The present study examined the associations between Chinese American mothers’ experiences of two types of stress (i.e., general/contextual stress and parenting stress) and their preschoolers’ socio-emotional and behavioral adjustment problems; in addition, the mediating roles of maternal psychologically controlling parenting and maternal warmth in these associations were assessed. Participants included 207 first-generation Chinese American mothers (*M*_age_ = 37.78 years, *SD*_age_ = 4.36) and their 3- to 6-year-old children (*M*_age_ = 4.50 years, *SD*_age_ = 0.90; 52% boys). Mothers reported on their levels of stress, psychologically controlling parenting, and warmth practices; teachers reported on child adjustment in the school setting. The results revealed that higher levels of general/contextual stress and parenting stress were each uniquely associated with more maternal psychologically controlling parenting practices, which in turn was associated with fewer socio-emotional and behavioral adjustment problems in children. Our findings can inform parenting intervention programs designed to improve Chinese American preschoolers’ adjustment.

## 1. Introduction

Preschool children who are socio-emotionally and behaviorally competent are more likely to develop positive peer and adult relationships [1] and are at lower risks for subsequent adjustment problems [2]. Thus, it is important to understand contributors to the socio-emotional and behavioral adjustment of young children. Children’s socio-emotional and behavioral adjustment include their pro-social and anti-social behaviors as well as problematic behaviors within the school setting [3]. This study used teachers’ reports on children’s socio-emotional and behavioral problems to reflect children’s adjustment in the school setting. Asian Americans are the fastest-growing immigrant group in the United States [4,5], with Chinese Americans being the largest subgroup. However, the socio-emotional and behavioral adjustment of Chinese American children remains understudied [6].

Parental stress has been noted as a significant contributor to preschoolers’ socio-emotional and behavioral adjustment because preschool children spend most of their time with their parents, which enhances the possibility of parents transmitting their feelings of stress to their children [7]. Also, parenting responsibilities can lead to high levels of stress, particularly during the preschool period [8,9]. Indeed, studies have shown that certain parental stress, such as parenting-specific stress, is associated with more child adjustment problems [10]. However, few studies have systematically examined different types of parental stress and their potentially unique associations with child adjustment difficulties [11]. According to Bornstein’s specificity principle [12], the development of specific characteristics in specific individuals is affected by specific experiences in specific ways. Therefore, it is necessary to consider specific family contexts indexed by different types of parental stress in relation to preschool children’s adjustment. Furthermore, much is unknown about the experience of stress among Chinese American mothers, who tend to be primary caretakers in these families, and its impact on their children’s socio-emotional and behavioral adjustment.

Another limitation in the literature is that the mechanisms through which parental stress may affect child outcomes remain unclear. According to Belsky’s process model of determinants of parenting [13] and Abidin’s parenting stress model [14], parental stress can negatively influence parenting practices, which in turn, may result in child adjustment problems [15]. However, this mediating process has not received much empirical attention [16,17], and it remains unclear how different parenting practices may be uniquely related to specific types of parenting stress and subsequent child adjustment [18].

We focused on two core dimensions of parenting practices as potential mediators of the association between parental stress and child socio-emotional and behavioral adjustment: control and warmth. Specifically, we examined maternal psychologically controlling parenting (PCP) and maternal warmth practices. PCP refers to parents’ attempts to control children’s psychological and emotional development by using guilt induction, shaming, and love withdrawal practices [19]. In general, PCP has been shown to contribute to negative child outcomes in diverse cultural settings, including Chinese and Chinese American families [20,21], but some researchers found no relation between certain types of PCP and child outcomes in both Chinese and European American families [22]. In addition, PCP may have different and more nuanced meanings in Chinese and Chinese American compared to Western families [23,24]. Specifically, parents from cultures that focus on interdependence among family members, such as China, may adopt the subtype of PCP, guilt induction, to guide their children to focus on the parents’ feelings and perspectives in order to help children develop empathy and connectedness with others [25].

Maternal warmth pertains to the quality of the affectional bond between mothers and their children as well as the physical and verbal behaviors through which mothers’ feelings are expressed [26]. Maternal warmth is associated with positive child adjustment across different cultural contexts [27]. However, the positive effect of warmth on child outcomes may be less robust in families with Asian backgrounds, possibly depending on how warmth is expressed in those families. For example, Chinese American parents tend to show warmth in more indirect ways compared to European American families [28].

In order to address the aforementioned gaps in the literature, the present study examined the relations between two types of parental stress, parenting practices, and child adjustment in Chinese American families.

### 1.1. General/Contextual Stress Versus Parenting Stress

Parents may experience stress of different nature and origin [29]. General/contextual stress is induced by stressors from a variety of sources in an individual’s life including work, marriage, finance, and environmental issues, whereas parenting stress originates from the role of being a parent [30] and the perceived accessibility and availability of resources for parenting relative to the demands of parenting [10,14]. Experiences of general/contextual stress and parenting stress may be different in two important ways. First, Lazarus [31] suggests that stress is not unidimensional but has qualitatively different subtypes including harm, threat, and challenge. While general/contextual stress, such as financial stress, may be more of a threat or harm or both, parenting stress may be more of a specific challenge. Second, researchers have argued that all parents experience parenting stress to some degree, regardless of the characteristics of parents and children, family socioeconomic status, and parents’ support networks [10]. However, general/contextual stress may not be experienced by all parents. For example, parents with higher socioeconomic status are less likely to experience financial stress than parents with lower socioeconomic status [32].

Parental general/contextual stress is associated with poorer child development [33,34,35,36]. For example, Duran et al. [33] found that increased family financial strain (an indicator of general/contextual stress) was negatively associated with delay of gratification among children from low socioeconomic backgrounds. Parents’ daily general stress was also positively associated with child psychosocial difficulties [36].

Parenting stress is also associated with both teacher-rated [8] and parent-rated [9,11,16,17] preschoolers’ adjustment problems. For instance, Huth-Bocks and Hughes [16] found that parenting stress was strongly associated with mother-reported child behavior problems in African American mother-child dyads. Similarly, Liu and Wang [9] reported that maternal parenting stress was directly associated with Chinese children’s internalizing and externalizing problem behaviors. Kochanova et al. [17] found a longitudinally positive association between parenting stress and child internalizing behaviors.

However, most previous research on parental stress and child adjustment focused on single types of stress (mostly parenting stress), whereas the simultaneous contribution of general/contextual stress and parenting stress has been largely neglected [17,37,38,39,40]. To our knowledge, only one study examined both stressors simultaneously and found that parenting stress was positively associated with child behavior problems whereas general/contextual stress was negatively associated with children’s theory of mind performance but not behavior problems in European American families [34]. In the present study, we examined general/contextual stress and parenting stress simultaneously and their unique associations with teacher-reported child socio-emotional and behavioral adjustment problems in Chinese American families. Based on the literature, we expected that both mothers’ perceived general/contextual stress and parenting stress would be positively associated with children’s negative adjustment.

### 1.2. The Mediating Roles of Parenting Practices

Scholars have suggested that parenting practices may serve as a potential mechanism through which parental stress affects child adjustment [14,15]. Negative parenting practices may result from both general/contextual stress and parenting stress. Specifically, parents’ allostatic response systems (i.e., systems that respond to physical, psychosocial, and environmental challenges; [41]) may become overburdened and dysregulated when trying to manage both the stress from normative challenges from various roles, such as the parenting role, and the stress unrelated to particular role challenges, such as economic adversity; in turn, the overburdened allostatic response systems may lead to maladaptive parenting practices [42].

Studies on the mediating role of parenting practices in the link between parental stress and child adjustment have yielded inconsistent findings. Some studies found that parenting practices, such as the use of discipline and display of approval [43] and responsiveness [44], mediated the link between parental stress and child adjustment outcomes including child self-control and behavior problems, whereas others found no mediating effect [8,11,16,33]. Furthermore, these previous studies only assessed either general/contextual stress or parenting stress. In this study, we focused on the potential mediating roles of parenting practices that tap into two core dimensions of parenting: control and warmth. Specifically, we focused on the roles of maternal PCP and maternal warmth practices in the relations between two types of parental stress (i.e., general/contextual stress and parenting stress) and child adjustment problems.

#### 1.2.1. Psychologically Controlling Parenting

PCP has been proposed to negatively affect child development by undermining children’s universal basic need for autonomy [21,45]. In general, PCP has been shown to be associated with child maladjustment across diverse cultural contexts, including in Chinese and Chinese American families [21,46,47]. However, researchers have argued for the consideration of specific cultural contexts in understanding the implications of parental control on child outcomes [20,48]. PCP may be less detrimental in societies where such practices fit the socialization goals of collectivistic familial contexts and, therefore, might be perceived as more normative and less maladaptive [21]. Indeed, there is some evidence supporting the culturally unique meanings and greater normativeness of certain subscales of PCP in Chinese cultures [22,49].

Scharf and Goldner [50] suggested that one predictor of PCP is stress from a variety of sources, including objective sources of stress (e.g., general/contextual stress) and factors within the parent (e.g., parenting stress). These authors suggested two ways through which stress can lead to PCP. First, stressful events can exhaust parental resources and force parents to adopt a quick mode of reaction, which might lead parents to adopt PCP practices in order to save time. Second, parents in stressful and challenging circumstances, such as those with fewer resources, may feel overwhelmed and powerless, resulting in more controlling parenting practices such as PCP. Moreover, parenting stress may result in more controlling parenting compared to general/contextual stress, because experiences of stress derived from the parenting role, for example, being worried about children’s adjustment problems, are more directly related to parent-child interactions, which may more strongly motivate parents to engage in controlling parenting practices to solve children’s adjustment problems.

Empirical studies have shown that parents with higher general/contextual stress tend to use more controlling parenting practices [51] and that parents with higher parenting stress are more likely to engage in authoritarian and coercive parenting practices [8,52,53,54], including PCP [9,55]. In turn, PCP has been generally found to be associated with greater internalizing and externalizing problems [9,10,56]. However, it should be noted that some studies found some forms of PCP, such as guilt induction, shaming, reciprocity, and social comparison, to be unrelated to Chinese children’s behavior problems [22]. In addition, one dimension of PCP, guilt induction, has been found to be associated with less bullying and aggressive behavior in Chinese American preschoolers [49]. In the current study, due to the inconsistent findings on the association between PCP and child adjustment, we did not propose a directional hypothesis but predicted that both types of parental stress would be indirectly related to child adjustment through maternal PCP, and this association would be stronger for parenting stress than general/contextual stress.

#### 1.2.2. Maternal Warmth

Another major dimension of parenting is warmth, which has been found to be associated with positive child adjustment, including fewer socio-emotional [57,58] and behavioral [27,59,60] adjustment problems. Maternal warmth practices were similarly associated with positive child outcomes across cultural contexts, including in Chinese and Chinese American families [58,61], although Chinese and Chinese American mothers express less direct and explicit forms of warmth towards their children compared to European American mothers [28,61,62].

Maternal warmth can be negatively affected by general/contextual stress [63] and parenting stress [8,11]. For example, household chaos [63], work stress [64], and parenting stress derived from parental distress, child characteristics, and parent–child dysfunctional interaction [8], and daily hassles [11], were negatively associated with maternal warmth, nurturing behaviors, and positivity. Moreover, maternal warmth has been found to mediate the negative associations between parental stress and child adjustment. Specifically, Jeon and Neppl [65] found that maternal positivity mediated the longitudinal association between economic pressure and child social competence from toddlerhood through the preschool years. However, the mediating role of maternal warmth in the relation between parenting stress and child adjustment is less supported. For example, Crnic et al. [11] found that the link between parenting daily hassles and child behavior problems was not mediated by parental positivity.

General/contextual stress may negatively impact maternal warmth more strongly than parenting stress because general/contextual stress represents broader concerns and may exhaust parental resources to a greater extent and negatively influence the emotional climate of warmth [66]. For example, Miao et al. [67] found that Chinese Canadian parents with higher levels of acculturation stress (a source of general/contextual stress) showed a decrease in positive parenting practices, including warmth, whereas stress from within the parent–child relationship (i.e., parenting stress) was associated with an increase in positive parenting practices. The authors explained the unexpected findings with measurement and operationalization considerations: the stress from within the parent–child relationship was measured by asking parents the intensity of conflict between parents and children, and parents may change their parenting practices into a more supportive way to resolve the conflict. Thus, specific parenting stress (e.g., conflicts between parents and children) may not always contribute to lower warmth. Given these findings, we hypothesized that general/contextual and parenting stress would be indirectly related to poorer child adjustment through less maternal warmth, and this association would be stronger for general/contextual stress than parenting stress.

### 1.3. The Present Study: Aims and Hypotheses

**Figure 1:**
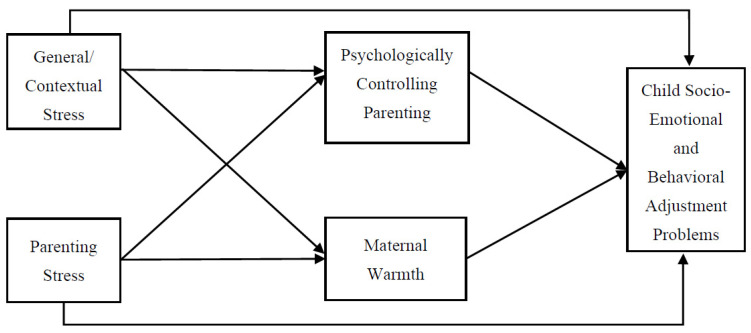
The conceptual mediation model for parental stress, parenting practices, and child adjustment problems. Note: Child age, child gender, maternal age, maternal education level, and maternal behavioral acculturation to the mainstream culture were included as the potential covariates in the model.

The present study had two aims (see Figure 1 for the conceptual model). The first aim was to examine Chinese American mothers’ perceived general/contextual stress and parenting stress in relation to their children’s socio-emotional and behavioral adjustment problems. We expected that general/contextual stress and parenting stress would be uniquely and positively associated with children’s socio-emotional and behavioral adjustment problems. The second aim of the current study was to investigate whether parenting practices (i.e., maternal PCP and warmth) would mediate the relations between the two types of parental stress and child adjustment problems. We hypothesized that: (a) mothers’ general/contextual stress and parenting stress would be positively associated with their PCP, and in turn, their preschool children’s adjustment problems, although we did not propose a directional hypothesis for the association between PCP and children’s adjustment problems; (b) the indirect effect of parenting stress on child adjustment problems through maternal PCP would be stronger than that of general/contextual stress; (c) mothers’ general/contextual stress and parenting stress would be negatively associated with their parental warmth, which in turn, would be associated with fewer adjustment problems in their children; and (d) the indirect effect of general/contextual stress on child adjustment problems through maternal warmth would be stronger than that of parenting stress.

The current study utilized cross-sectional data to investigate the links between maternal stress, parenting practices, and child adjustment problems. Despite the limitations of cross-sectional data when testing mediation models [68], the indirect associations proposed in the present study were informed by both theoretical frameworks and empirical evidence. Given the novelty of the data herein, the findings from these cross-sectional data could serve as an initial step in understanding the potential mechanisms linking different types of parental stress and child adjustment outcomes.

Maternal age and education have been found to be related to parenting practices [61,69] and child age and gender and maternal behavioral acculturation to the mainstream culture have been found to be related to parenting practices and child adjustment [70,71,72,73]; therefore, these variables were examined as potential covariates in the current study.

## 2. Method

### 2.1. Participants

Participants included 207 first-generation Chinese American mothers (*M*_age_ = 37.78 years, *SD*_age_ = 4.36) and their 3- to 6-year-old children (*M*_age_ = 4.50 years, *SD*_age_ = 0.90, 52% boys) living in the Maryland/Washington D. C. metropolitan area in the United States. Mothers were born in mainland China (82%), Hong Kong (3%), Taiwan (14%), or other countries (1%). Mothers had lived in the United States for an average of 10.57 years (*SD* = 6.06 years). Most of their children were born in the United States (90%). Most mothers were married (98%) to a partner who also identified as ethnically Chinese, and 94% of mothers had at least a college degree or higher education.

### 2.2. Procedure

Participants were recruited from preschools, daycare centers, churches, and supermarkets. Mothers’ informed consent and children’s assent were obtained before data collection. Trained bilingual research assistants collected the data during home visits. Mothers completed the questionnaires about their perceived stress and parenting practices in their preferred language (simplified or traditional Chinese or English). With the parents’ permission, the teachers of the participating children were asked to report on the children’s socio-emotional and behavioral problems in the school setting. Ethical approval for the study was obtained from the University institutional review board of the University of Maryland, Baltimore County.

### 2.3. Measures

#### 2.3.1. General/Contextual Stress

The Hassles and Uplifts Scale (HUS) [74] was used to assess mothers’ perceived general/contextual stress. This measure consists of 53 items that measure stress appraisals across a variety of contexts (e.g., work, personal life, family, finances). We excluded one item about children to avoid content overlap with the parenting stress variable. Mothers rated each item on how much of a hassle each situation was on a 4-point scale, ranging from 0 (none) to 3 (a great deal). Sample items include, “Fellow workers,” “Recreation and entertainment outside the home,” “Your parents or parents-in-law,” and “Enough money for necessities.” The final general/contextual stress score was the sum of the 52 items, with higher scores indicating higher general/contextual stress. The reliability of the HUS in the current study was good (Cronbach’s α = 0.95).

#### 2.3.2. Parenting Stress

The Parenting Daily Hassles scale (PDH) [75] was used to assess mothers’ perceived parenting stress. The PDH consists of 20 items that measure parental perceptions about minor daily hassles and inconveniences pertaining to parenting (e.g., “Kids are difficult to manage in public places”). Mothers rated each item for how hassled they felt by the event (i.e., intensity) on a 5-point scale, ranging from 1 (no hassle) to 5 (big hassle). The final parenting stress score was the sum of the 20 items, with higher scores indicating higher parenting stress. The reliability of the PDH in the current study was good (Cronbach’s α = 0.91).

#### 2.3.3. Psychologically Controlling Parenting

The Psychological Control and Overprotective/Intrusive Measure (PCOIM) [76] was used to measure maternal PCP. The PCOIM includes 18 items that measure maternal PCP. A sample item for the PCP is, “I ignore my child when he/she tries to get attention.” Mothers rated each item on a 5-point scale ranging from 1 (never) to 5 (always). The final score was the sum of the 18 items, with higher scores representing higher levels of maternal PCP. The reliability of the PCP in the current study was good (Cronbach’s α = 0.84).

#### 2.3.4. Maternal Warmth

The warmth subscale of the Parenting Styles and Dimensions Questionnaire (PSDQ) [77] was used to measure maternal warmth. This warmth subscale is comprised of seven items. A sample item is, “Gives comfort and understanding when child is upset.” Mothers rated each item based on a 5-point scale, ranging from 1 (never) to 5 (always). The final score was the sum of the seven items, with higher scores representing higher levels of maternal warmth. The reliability of the warmth subscale of the PSDQ in the current study was adequate (Cronbach’s α = 0.78).

#### 2.3.5. Child Socio-Emotional and Behavioral Adjustment Problems

The Strengths and Difficulties Questionnaire for the teachers (SDQ-T) [78] was used to measure child socio-emotional and behavioral adjustment problems. The SDQ-T includes 20 items that measure the child’s problems with peers (e.g., “Rather solitary, prefers to play alone”), emotional symptoms (e.g., “Many worries or often seems worried”), conduct problems (e.g., “Often fights with other children or bullies them”), and hyperactivity/inattention (e.g., “Restless, overactive, cannot stay still for long”). Each of the four subscales has five items. Teachers rated each item on a 3-point scale ranging from 0 (not true) to 2 (certainly true). A total socio-emotional and behavioral adjustment problem score was created by summing all 4 subscales (peer problems, emotional symptoms, conduct problems, and hyperactivity/inattention), with higher scores representing more socio-emotional and behavioral difficulties. The reliability of the SDQ-T in the current study was good (Cronbach’s α = 0.81).

#### 2.3.6. Maternal Behavioral Acculturation towards the U.S. Mainstream Culture

In order to control for maternal acculturation level, the Cultural and Social Acculturation Scale (CSAS) [79] was used to measure maternal behavioral acculturation toward the mainstream American culture. The CSAS includes 11 items reflecting individuals’ behavioral cultural orientation to the mainstream culture in the domains of social relationships, English language use, and American living styles. A sample item includes, “How often do you read English novels or magazines?” The items were rated on a scale of 1 (almost never) to 5 (almost every day). A sum score of maternal behavioral acculturation toward the mainstream culture was created, with higher scores representing more behavioral acculturation towards the mainstream culture. The reliability in the current study was adequate (Cronbach’s α = 0.76).

#### 2.3.7. Data Analysis

First, descriptive statistics of and the zero-order correlations among the study variables were examined using SPSS 27.0. Second, a multiple regression model in which child adjustment problems were regressed on both general/contextual stress and parenting stress was conducted to examine the associations between the two types of stress and child adjustment problems. Third, path analysis was conducted using the “lavaan” package in R studio to examine the mediating roles of maternal PCP and maternal warmth between the two types of parental stress and child socio-emotional and behavioral adjustment problems. We examined the correlations between potential covariates and the mediators and the outcome variables. The demographic variables significantly correlated with mediators and/or outcomes were included as covariates in the regression and path analyses.

The rate of item-level missing data was smaller than 6%, and the data were missing completely at random based on Little’s Missing Completely at Random test: χ^2^(4793, *N* = 207) = 4921.47, *p* = 0.096 [80]. The Mardia’s Multivariate Normality (MVN) test [81] revealed that the multivariate normality of the variables of interest was violated. Therefore, we used the robust maximum likelihood (MLR) estimation to correct for the bias and produce more accurate estimates of parameters. The model fit was evaluated by χ^2^ statistics, comparative fit index (CFI), Tucker-Lewis index (TLI), root mean square error of approximation (RMSEA), and standardized root mean square residual (SRMR). Statistically non-significant χ^2^ value suggests a good model fit. The model fit was deemed acceptable with CFI and TLI values above 0.90, RMSEA below 0.08, and was deemed good with CFI and TLI values above 0.95, RMSEA below 0.05, and SRMR below 0.08 [82,83]. The indirect effects were evaluated using a bootstrapping approach with 95% bias-corrected bootstrapped confidence intervals (CI) based on 1000 bootstrap samples [84].

## 3. Results

### 3.1. Preliminary Analyses

Descriptive statistics and correlations are presented in Table 1. The zero-order correlations showed that neither general/contextual stress nor parenting stress was correlated with children’s socio-emotional and behavioral adjustment problems. Moreover, general/contextual stress was significantly correlated with higher maternal PCP and lower maternal warmth, whereas parenting stress was only significantly correlated with higher maternal PCP. Finally, neither maternal PCP nor maternal warmth was correlated with children’s socio-emotional and behavioral adjustment problems. However, there was a significant and negative partial correlation between maternal PCP and children’s adjustment problems (*r* = −0.16, *p* = 0.023), while controlling for the effect of parenting stress. In terms of the potential covariates, child age and gender were each significantly and negatively correlated with child adjustment problems; maternal education was significantly and negatively correlated with parenting stress and child adjustment problems; maternal behavioral acculturation to mainstream culture was significantly and positively correlated with maternal warmth. Maternal age was not correlated with any mediators or the outcome variable and was not included in the subsequent analyses.

### 3.2. Association between Parental Stress and Child Adjustment

Child age, child gender, maternal education, and maternal behavioral acculturation to mainstream culture were included as covariates in the multiple regression model. The regression model revealed that neither general/contextual stress (*b* = 0.01, *p* = 0.485) nor parenting stress (*b* = 0.02, *p* = 0.531) was significantly related to children’s socio-emotional and behavioral adjustment problems (see Table 2).

### 3.3. The Mediating Roles of Parenting Practices

**Figure 2:**
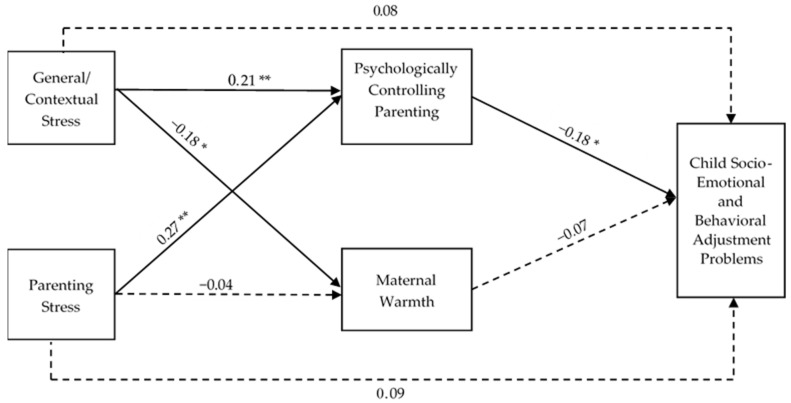
The mediation model for parental stress, parenting practices, and child adjustment problems. Note: Child age, child gender, maternal education level, and maternal behavioral acculturation to mainstream culture were included as covariates in the model. Standardized regression coefficients were indicated. Solid lines represent significant paths and dashed lines represent non-significant paths. * *p* < 0.05, ** *p* < 0.01.

A path model was conducted to examine the mediating roles of maternal PCP and maternal warmth practices in the relations between two types of parental stress and children’s socio-emotional and behavioral adjustment problems (see Figure 2). Child age, child gender, maternal education level, and maternal behavioral acculturation to mainstream culture were treated as covariates. All covariates and exogenous variables were allowed to covary, and the residuals of mediators were allowed to covary. The goodness-of-fit indices indicated a good model fit: χ^2^(1, *N* = 207) = 1.00, *p* = 0.606, CFI = 1.00, TLI = 1.04, SRMR = 0.01, RMSEA = 0.00, 90% CI [0.00, 0.00]. The model explained 20.2% of the variance in maternal PCP, 11.6% of the variance in maternal warmth, and 11.3% of the variance in children’s socio-emotional and behavioral adjustment problems.

Neither general/contextual stress (*b* = 0.02, *SE* = 0.02, *p* = 0.380, 95% CI [−0.023, 0.060]) nor parenting stress (*b* = 0.03, *SE* = 0.03, *p* = 0.245, 95% CI [–0.023, 0.092]) was directly associated with child adjustment problems. Higher levels of general/contextual stress (*b* = 0.09, *SE* = 0.03, *p* = 0.009, 95% CI [0.022, 0.153]) and parenting stress (*b* = 0.19, *SE* = 0.06, *p* = 0.001, 95% CI [0.079, 0.295]) were each uniquely associated with higher levels of maternal PCP. The difference between the magnitudes of these two associations was not significant. Higher levels of maternal PCP, in turn, were associated with fewer children’s socio-emotional and behavioral adjustment problems (*b* = −0.10, *SE* = 0.04, *p* = 0.018, 95% CI [−0.180, −0.017]). Both general/contextual stress (*ab* = −0.01, *SE* = 0.01, 95% CI [−0.021, −0.001]) and parenting stress (*ab* = −0.02, *SE* = 0.01, 95% CI [−0.043, −0.003]) had a significant and negative indirect effect on children’s adjustment problems through maternal PCP.

Maternal warmth was significantly and negatively associated with general/contextual stress (*b* = −0.03, *SE* = 0.01, *p* = 0.019, 95% CI [−0.056, −0.005]) but not parenting stress (*b* = −0.01, *SE* = 0.02, *p* = 0.636, 95% CI [−0.059, 0.036]). However, maternal warmth was not significantly associated with children’s socio-emotional and behavioral adjustment problems. Therefore, the indirect effects of general/contextual stress and parenting stress on children’s adjustment through maternal warmth practices were not examined.

## 4. Discussion

Chinese American mothers may experience different types of stress that contribute to their parenting practices and children’s socio-emotional and behavioral adjustment in various ways. The current study examined the unique associations between two types of parent-experienced stress (i.e., general/contextual stress and parenting stress) and Chinese American children’s adjustment problems as well as the mediating roles of two parenting practices (i.e., maternal PCP and maternal warmth) in these associations.

Contrary to our first hypothesis and previous findings indicating that parental stress is associated with more child adjustment problems [11], our results revealed that neither general/contextual stress nor parenting stress was directly associated with teacher-rated adjustment problems in Chinese American preschool children. The inconsistent findings may be due to the different approaches used to measure children’s adjustment problems. For example, Crnic and colleagues [11] used parent-reported child behavioral problems, which may capture child problems in the home setting, while our study used teacher-reported child adjustment problems that reflected child problems in school. Moreover, parents’ perceived experiences of stress and their own perceptions of their children’s behaviors are more likely to be associated with each other due to shared- or common-method variance [85]. Thus, our use of separate reporters of parental stress and child adjustment is a more robust test of the direct association between these two constructs. Another possible explanation is that Chinese American mothers in our sample did not experience very high levels of stress on average (*M*_general/contextual stress_ = 28.5, possible range = 0 to 156; *M*_parenting stress_ = 43.3, possible range = 20 to 100), so the stress experienced by mothers might not be sufficiently salient to be directly associated with child adjustment.

As expected, both general/contextual stress and parenting stress were positively associated with higher levels of maternal PCP, although the magnitudes of the two associations were not significantly different. This finding indicates that both perceptions of general/contextual stress and perceived stress pertaining to the parenting role specifically, such as being overwhelmed by children’s behaviors [50], can equally elicit maternal engagement in more controlling practices.

Interestingly, Chinese American children of mothers who reported more PCP showed fewer adjustment problems in the school setting. This finding suggests that the relation between PCP and child adjustment problems might be dependent on the cultural context. In independence-oriented cultures that value individualism, such as in European American families, PCP practices may be perceived as parental rejection and impede positive development in children. In contrast, in interdependence-oriented cultures, such as the Chinese culture, PCP has been found to be perceived as more normative and less maladaptive [22]. Children, especially young children, may be more likely to interpret PCP practices as parental concern for their well-being rather than parental rejection, which may buffer them against the negative effects of PCP [9].

Indeed, our finding is consistent with some previous research in which maternal PCP was found to be less detrimental to preschool-aged children because of the better fit with the interdependent familial socialization goals of first-generation Chinese American families [49]. Furthermore, young Chinese American children who are exposed to maternal PCP in the family context may learn to be more compliant and be oriented towards fulfilling others’ needs in order to receive approval, avoid disappointing others, and maintain social harmony in different contexts, such as the school context. In fact, while some forms of maternal PCP, such as love withdrawal, have been found to be associated with child behavior problems longitudinally, other forms, such as guilt induction, were found to be associated with fewer behavior problems in young children over time [49]. Parents’ practices of inducing mild guilt by illustrating the consequences of a child’s behaviors to their parents could improve the child’s understanding of others’ feelings [22]. It shoud be noted that the mean level of maternal engagement in PCP practices in the current study was not high (*M_PCP_* = 39.1, possible range = 18 to 90). Thus, mothers may not have enaged in sufficient levels of PCP practices to have a negative impact on their children’s adjustment.

Importantly, it needs to be emphasized that although the results showed that Chinese American mothers experiencing higher levels of stress engaged in more PCP, which in turn, contributed to fewer child adjustment problems, these findings do not suggest that stress itself is beneficial to child adjustment. Rather, as discussed above, the specific cultural context and levels at which PCP practices are used, as well as the nature of these PCP practices, need to be considered. In addition, as expected, the indirect effect of parenting stress on child adjustment problems through maternal PCP was larger than that of general/contextual stress. However, although the confidence intervals of the indirect effects of both general/contextual stress and parenting stress did not include zero, the upper limits were close to zero; therefore, future research is needed to replicate the results.

Our hypotheses regarding the two types of parental stress and maternal warmth were partially supported. Specifically, mothers who experienced higher levels of general/contextual stress reported lower levels of warmth; however, mothers’ perceived levels of parenting stress were not associated with maternal warmth, indicating the unique contributions of specific types of stress to specific parenting practices [12]. Compared to stress solely caused by the parenting role, general/contextual stress, including financial stress [33], household chaos [63], and work stress [64], may be more likely to be perceived by parents as a threat or harm [31] and drain parental energy to a greater extent [66]. Thus, these parents may be less motivated or resourced to express warmth. Maternal warmth was not associated with child adjustment problems in our sample, which is contrary to prior longitudinal study findings [27,57,60]. This inconsistency may be due to the different research designs used across studies. Specifically, this study used a cross-sectional design that only allowed us to examine the short-term effects of warmth, whereas in previous longitudinal studies, maternal warmth was found to have a long-term effect on children’s emotional adjustment [57] and externalizing problems [60]. This inconsistency may also reflect differences in previous studies’ examinations of concurrent versus longitudinal associations between maternal warmth and child adjustment. The effect of decreased parental warmth may gradually unfold and negatively influence children’s adjustment over time. Moreover, some research found that parental warmth was not related to either Chinese children’s externalizing problems or internalizing problems [86].

## 5. Conclusions

### 5.1. Limitations and Future Directions

Several limitations of the current study should be noted. First, because of the cross-sectional research design of the current study, no claims about causal or temporal relations between stress predictors, maternal PCP, maternal warmth, and child adjustment problems can be made. In fact, children with adjustment difficulties may evoke more controlling or less warm parenting practices [87,88] and lead to greater experiences of stress for their parents [89,90]. However, the current study serves as a useful initial step for further research because the study followed and met the six conditions of Pieters’ guidelines on conducting a meaningful mediation analysis [91]. Specifically, our study had: (a) a solid theoretical basis for the proposed paths’ directionality, (b) reliability of measures, (c) controlling significant confounding variables, (d) mediators and outcomes being conceptually and empirically distinguishable, (e) sufficient statistical power to identify true non-null effects, and (f) statistically significant indirect effects. Future longitudinal research and experimental designs should be employed to examine the temporal and causal associations among these variables as we proposed.

Second, we relied on mothers’ self-reports of stress and parenting practices, which may not accurately reflect their actual stress level and parenting practices, and may increase measurement bias and result in shared-method variance [85]. Future studies should consider spousal reports of parental stress and observational assessments of parenting practices. Finally, our sample was comprised of Chinese Americans predominantly from middle-class backgrounds residing in the Maryland/Washington metropolitan area. Thus, generalizations of our results to Chinese American populations with other sociodemographic characteristics and those residing in other parts of the United States should be made cautiously.

### 5.2. Implications

Previous studies on parent-perceived stress and child outcomes have rarely investigated general/contextual stress and parenting stress simultaneously, but rather focused on one specific type of stress (e.g., parenting stress or financial stress) at a time. The current study contributed to the literature by examining the concurrent and unique roles of both general/contextual stress and parenting stress in child adjustment problems. This study also advanced our knowledge of potential underlying mechanisms between parent-perceived stress and child adjustment by investigating two core dimensions of parenting (i.e., control and warmth) and by bridging these theoretical frameworks [13,14,15] with the specificity principle [12] in Chinese American families.

General/contextual stress and parenting stress were found to be differentially associated with psychologically controlling and warm parenting practices, which in turn, had unique associations with Chinese American children’s socio-emotional and behavioral adjustment problems. This finding supports the specificity principle [12] by demonstrating that parental stress experienced in specific contexts can contribute to children’s adjustment through specific parenting practices. We complemented the common assumption that only overall stimulation or a shared experience affects overall development and facilitated our knowledge of the development of specific characteristics in specific individuals. Our findings can also inform the development of more effective and culturally competent parenting intervention programs. Specifically, intervention programs should make more efforts to provide specific coping strategies appropriate to the specific types of stress being experienced by Chinese American parents.

In conclusion, the current study revealed unique associations between specific types of stress experienced by Chinese American mothers and their parenting practices and subsequent socio-emotional and behavioral adjustment problems in their preschool children. These findings provided empirical evidence that parenting practice is a critical proximal process linking maternal stress and child adjustment problems in Chinese American families and set the foundation for future longitudinal research.

## Figures and Tables

**Table 1:** Descriptive statistics and correlations for all study variables.

Variable	*M*	*SD*	1	2	3	4	5	6	7	8	9	10
1. GCS	28.5	20.4	-									
2. Parenting stress	43.3	12.6	0.51 ***	-								
3. Maternal PCP	39.1	8.6	0.34 ***	0.38 ***	-							
4. Maternal warmth	29.8	3.4	−0.19 **	−0.12	−0.09	-						
5. CSEBAP	6.0	4.7	0.11	0.09	−0.11	−0.08	-					
6. Child age	4.5	0.9	−0.09	−0.05	0.12	−0.05	−0.15 *	-				
7. Child gender ^a^	-	-	−0.03	0.07	−0.03	0.01	−0.17 *	−0.11	-			
8. Maternal age	37.8	4.4	−0.05	−0.12	0.07	0.04	0.05	0.32 ***	−0.24 ***	-		
9. Maternal education	-	-	−0.11	−0.19 **	−0.08	0.08	−0.16 *	0.05	−0.00	0.17 *	-	
10. MBAM	33.5	7.0	0.01	0.01	0.07	0.26 ***	−0.12	0.04	−0.10	0.20 **	0.40 ***	-

Note: *N* = 207. GCS = general/contextual stress; PCP = psychologically controlling parenting; CSEBAP = child socio-emotional and behavioral adjustment problems; MBAM = maternal behavioral acculturation to mainstream culture. ^a^ 1 = boys, 2 = girls. * *p* < 0.05, ** *p* < 0.01, *** *p* < 0.001.

**Table 2:** Regression: associations between parental stress and child adjustment.

Variables (*R*^2^ = 0.30)	*b* (*SE*)	*f^2^*
General/contextual stress	0.01 (0.02)	0.002
Parenting stress	0.02 (0.03)	0.002
Child age	−0.79 * (0.36)	0.02
Child gender	−1.83 ** (0.65)	0.04
Maternal education	−0.69 (0.50)	0.01
Maternal behavioral acculturation to mainstream culture	−0.06 (0.05)	0.01

Note: *N* = 207. * *p* < 0.05, ** *p* < 0.01.

## Data Availability

Data are available through the authors at reasonable request.
